# Comparative Economic Evaluation of *Haemophilus influenzae* Type b Vaccination in Belarus and Uzbekistan

**DOI:** 10.1371/journal.pone.0021472

**Published:** 2011-06-24

**Authors:** Ulla K. Griffiths, Andrew Clark, Veronika Shimanovich, Irina Glinskaya, Dilorom Tursunova, Lucia Kim, Liudmila Mosina, Rana Hajjeh, Karen Edmond

**Affiliations:** 1 Hib Initiative, London School of Hygiene and Tropical Medicine, London, United Kingdom; 2 Republican Centre for Hygiene, Epidemiology and Public Health, Minsk, Belarus; 3 Minsk City Centre for Hygiene and Epidemiology, Minsk, Belarus; 4 Ministry of Health, Tashkent, Uzbekistan; 5 WHO Regional Office for Europe, Copenhagen, Denmark; 6 Center for Disease Control, Atlanta, Georgia, United States of America; University of Witwatersrand, South Africa

## Abstract

**Background:**

Hib vaccine has gradually been introduced into more and more countries during the past two decades, partly due to GAVI Alliance support to low-income countries. However, since Hib disease burden is difficult to establish in settings with limited diagnostic capacities and since the vaccine continues to be relatively expensive, some Governments remain doubtful about its value leading to concerns about financial sustainability. Similarly, several middle-income countries have not introduced the vaccine. The aim of this study is to estimate and compare the cost-effectiveness of Hib vaccination in a country relying on self-financing (Belarus) and a country eligible for GAVI Alliance support (Uzbekistan).

**Methods and Findings:**

A decision analytic model was used to estimate morbidity and mortality from Hib meningitis, Hib pneumonia and other types of Hib disease with and without the vaccine. Treatment costs were attached to each disease event. Data on disease incidence, case fatality ratios and costs were primarily determined from national sources. For the Belarus 2009 birth cohort, Hib vaccine is estimated to prevent 467 invasive disease cases, 4 cases of meningitis sequelae, and 3 deaths, while in Uzbekistan 3,069 invasive cases, 34 sequelae cases and 341 deaths are prevented. Estimated costs per discounted DALY averted are US$ 9,323 in Belarus and US$ 267 in Uzbekistan.

**Conclusion:**

The primary reason why the cost-effectiveness values are more favourable in Uzbekistan than in Belarus is that relatively more deaths are averted in Uzbekistan due to higher baseline mortality burden. Two other explanations are that the vaccine price is lower in Uzbekistan and that Uzbekistan uses a three dose schedule compared to four doses in Belarus. However, when seen in the context of the relative ability to pay for public health, the vaccine can be considered cost-effective in both countries.

## Introduction

*Haemophilus influenzae* type b (Hib) is an encapsulated, Gram-negative coccobacillus that can cause meningitis, pneumonia and a number of rarer forms of disease, such as epiglottitis, septicaemia and cellulitis, primarily in children less than five years [1]. Survivors of Hib meningitis risk severe lifelong disabilities, such as deafness and neurological disorders [2]. A Hib conjugate vaccine has been available since the early 1990s and has led to virtual elimination of the disease in countries with sustained high vaccination coverage [3], [4].

In Eastern Europe and Central Asia, Hib vaccine uptake has been slow, primarily due to uncertainty about Hib disease burden and because the vaccine is considerably more expensive than traditional childhood vaccines [5]. For the nine poorest countries in the regions, including Uzbekistan, Hib vaccine introduction has been facilitated by funding from the GAVI Alliance. The other eight European and Central Asian countries that have received Hib vaccine from the GAVI Alliance are Albania, Armenia, Azerbaijan, Bosnia & Herzegovina, Georgia, Moldova, Kyrgyzstan and Tajikistan. The support to Uzbekistan began in March 2009 and is scheduled to end in 2015, at which point the Government needs to decide whether it wants to continue using the vaccine and if so, how it will be financed. In Belarus, Hib vaccination was started by the local Government of Minsk city in 2008 covering around one-fifth of the birth cohort. The national Government is currently considering whether to expand it to the rest of the country.

The aim of this study is to estimate and compare the cost-effectiveness of Hib vaccination in a country relying on self-financing (Belarus) and in a country eligible for GAVI Alliance support (Uzbekistan). The Governments of both countries requested the analyses from the WHO with the purpose of using the evidence to guide their decision making on the vaccine.

## Methods

### Hib disease definitions

Hib disease was categorised as pneumonia, meningitis, and “non-pneumonia-non-meningitis” (NPNM), replicating groupings used by the Global Burden of Hib and Pneumococcal disease project [6], [7]. Pneumonia was defined as any case of pneumonia recorded by the Ministry of Health surveillance databases in Uzbekistan and Belarus, regardless of radiological confirmation. Meningitis was defined as any case of inpatient hospital meningitis. NPNM included other specific diseases in children that can be caused by the Hib bacterium, such as cellulitis, osteo myelitis, septic arthritis and epiglottitis.

### Decision analytic model structure

**Figure 1:**
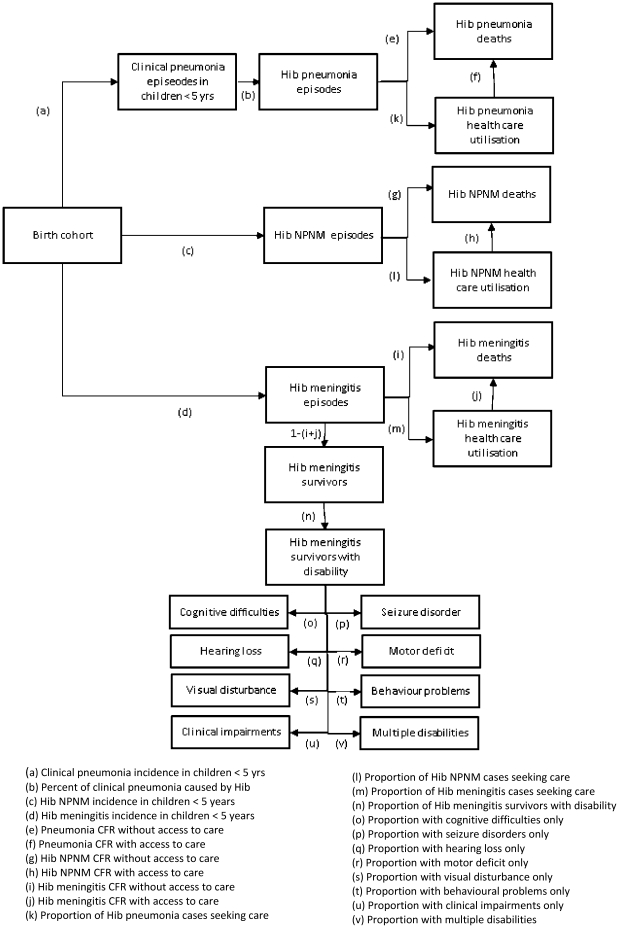
Hib disease model structure.

A static, compartmental cohort model was used to estimate Hib disease events for children of the 2009 birth cohorts in the two countries. The model is illustrated in Figure 1. Projected numbers of person-years lived between 1 and 59 months were multiplied by disease incidence rates to estimate Hib cases in each cohort. An all-cause pneumonia incidence rate was used to calculate total pneumonia cases and it was assumed that a proportion of these were caused by Hib. Hib meningitis and Hib NPNM cases were calculated directly from aetiology specific incidence rates. A proportion of cases were assumed to seek health care and each of these incurred an average treatment cost. Hib deaths were estimated from case fatality ratios (CFRs) and a risk of permanent disability was applied to all survivors of Hib meningitis and classified according to type.

The impact of Hib vaccine was estimated as the difference between scenarios with and without the vaccine. In the vaccine scenario, numbers of cases were reduced by dose-specific vaccination coverage and dose-specific vaccine efficacy. Incremental cost-effectiveness ratios (ICERs) were calculated by dividing the difference in expected costs between the two scenarios with the difference in expected health effects, expressed as cases, deaths and lost Disability Adjusted Life Years (DALYs). The analysis was undertaken from a societal perspective; including costs incurred by the Governments, the GAVI Alliance and households. While the Government health sector is dominant in both countries with very limited private health services available, it is common practice in Uzbekistan that patients pay a proportion of drug costs when accessing public services and these were included in the analysis. Transport and time costs were excluded as we did not conduct patient interviews as part of the study.

Future costs and effects were discounted by 3% per year [8]. Treatment costs were estimated in 2009 US$ using exchange rates of 2,793 Belarusian rubles and 1,464 Uzbek som to one US$. The model was developed in Microsoft Excel. Model input parameter values are summarised in Table 1 and justifications for these given below.

**Table 1:** Parameter values used in the base-case analysis.

Parameter name	Belarus	Uzbekistan	SourcesBelarus/Uzbekistan
2009 live births	96,337	558,459	[17]
2009 life expectancy at birth (years)	69	68	[17]
2009 vaccine coverage of third DTP dose	96%	98%	[30]
Incidence per 100,000 children < 5 yrs:			
All-cause clinical pneumonia	2,302	2,277	Belarus/Uzbek MOH
Hib NPNM	6.15	10.65	[19], [20]
Hib meningitis	10.1	18.7	[12], [13]/[16]
Proportion of clinical pneumonia due to Hib	5%	5%	[7]
Proportion of meningitis cases with disability	12%	14.5%	Minsk City surveillance/[2]
Case fatality ratios:			
Hib pneumonia w/o access to care	NA	10%	NA/Assumption
Hib pneumonia with access to care	0.3%	1.07%	MOHs routine surveillance
Hib NPNM w/o access to care	NA	78%	NA/Assumption
Hib NPNM with access to care	2.5%	12%	Assumptions
Hib meningitis w/o access to care	NA	100%	NA/Assumption
Hib meningitis with access to care	3.2%	15%	[13]/[23]
Treatment utilization			
Percent of pneumonia cases seeking care	100%	68%	[10]/[9]
Percent of NPNM cases seeking care	100%	68%	[10]/[9]
Percent of meningitis cases seeking care	100%	68%	[10]/[9]

### Health care utilization

As seen in Figure 1, CFRs are assumed to vary with health care utilisation, implying that the risk of death is higher if no formal health care services are accessed. Similarly, when incidence rate calculations are based on data from hospital surveillance only, it is necessary to adjust these as cases would inevitably be missed in settings with limited access to care. In Uzbekistan, a 2006 Multiple Indicator Cluster Survey reported that of children aged 0–59 months with pneumonia symptoms during the two weeks preceding the survey, only 68% were taken to an appropriate provider [9]. We used this figure to adjust CFRs and hospital disease incidence rates for all types if Hib disease.

Balabanova *et al*. illustrated that Belarus has the highest health service utilization out of eight former Soviet Union countries[10], and the country also has the largest number of doctors and nurses per 1,000 population within central- and Eastern Europe [11]. In particular, children's health is monitored closely with general check-ups by all main specialists annually until 18 years of age [11]. Therefore, we did not adjust hospital incidence rates and CFRs for limited access to care in Belarus.

### Hib disease incidence parameters

#### Hib meningitis

Meningitis is the most studied type of Hib disease for three key reasons: it is the most severe form of Hib disease, it has a relatively straightforward clinical diagnosis, and it is the type of Hib disease that can be most easily diagnosed in a laboratory (from cerebrospinal fluid (CSF) of patients with clinical symptoms). Hence, when assessing Hib disease burden, meningitis is often the preferred starting point.

In Belarus, population-based childhood bacterial meningitis surveillance was in place at Minsk City Children's Infectious Disease Hospital (MCCIDH) during 2002–2007 [12], [13]. During the six-year period, 175 purulent meningitis cases were detected in children < 5 years and a bacterial pathogen was identified for 87 of these, giving an average bacterial meningitis incidence rate of 31 per 100,000 children < 5 years (Table 2). 30 of the cases were confirmed as Hib, generating an incidence of 5 per 100,000 children, with annual rates between 1 and 10. However, since 88 of the purulent cases had no confirmed pathogen, this is likely to be an underestimate of the true number. According to WHO guidelines [14] and following methods used in other bacterial meningitis aetiology studies [15], culture negative cases should be allocated to bacterial pathogens according to their proportion of confirmed cases. Hence, we apportioned 34% of the non-confirmed cases to Hib and estimated an *adjusted* Hib meningitis incidence rate of 10.8 per 100,000 children < 5 years, with a range between 7.4 and 15.1.

**Table 2:** Reported purulent* meningitis cases in children less than 5 years in Minsk city, Belarus.

Year	Number of confirmed Neisseria meningitis cases	Number of confirmed Hib cases	Number of confirmed Streptococcus pneumoniae cases	Number of confirmed other bacteria	Number of culture negative cases	Number of purulent cases*	Bacterial meningitis incidence per 100,000 children < 5 years**
**2002**	3	2	1	0	12	18	24.94
**2003**	7	6	1	0	11	25	33.57
**2004**	10	5	1	0	12	28	36.54
**2005**	9	7	3	0	14	33	42.13
**2006**	7	1	3	1	25	37	45.69
**2007**	6	5	1	1	6	19	22.27
**2008**	3	4	0	0	8	15	16.37
**TOTAL**	**45**	**30**	**10**	**2**	**88**	**175**	**31.28**

*Purulent meningitis is defined as visibly turbid or cloudy OR WCC > 100.

**Incidence rates are calculated from the following under-five population in Minsk city: 2002: 72,168, 2003: 74,469, 2004: 76,623, 2005: 78,323, 2006: 80,985, 2007: 85,335, 2008: 91,623.

Since no population based meningitis surveillance studies have been conducted in Uzbekistan, we used routine surveillance data for our estimates. 234 hospitals reported 5,245 clinical meningitis cases in children < 5 years in 2007, giving an incidence of 166 per 100,000 children. We adjusted this estimate upwards by 32% to account for limited access to care, arriving at 219 per 100,000 children. The proportion of meningitis caused by Hib was approximated from a study conducted by the Uzbek Ministry of Health and the US Naval Medical Research Unit no. 3 in Egypt[16]. 165 CSF samples from children with clinical meningitis admitted to hospitals in Tashkent and Samarkand during 2002–2004 were tested using Polymerase Chain Reaction and 61 were found to be purulent with nine of them confirmed as Hib[16]. The *adjusted* number is thus 14 cases, equivalent to 8.5% of all the cases tested. We therefore estimated a Hib meningitis incidence of 18.7 per 100,000 children < 5 years (8.5% of 219).

For comparison, the Hib Global Burden of Disease (GBD) project estimated a Hib meningitis incidence rate of 16 per 100,000 children < 5 years for the WHO European region [6]. We used this estimate for in both countries in the sensitivity analyses.

#### Hib pneumonia

All-cause pneumonia incidences were determined from routine surveillance systems in both countries. In Belarus, 10,402 pneumonia cases in children less than five years were reported during 2008, giving an annual incidence of 2,302 per 100,000 children, based on an under-five population of 451,960[17]. 84% of cases were reported from hospitals and the remaining from outpatient clinics. In Uzbekistan, 20,014 pneumonia cases were reported in 2007, giving an incidence of 633 per 100,000 children < 5 years (under-five population is 3,162,151)[17]. 230,201 acute respiratory infections (ARI) cases were reported the same year, 15% of which were hospitalised. When hospitalised ARI cases are included, the incidence rate increases to 1,724 per 100,000. After adjusting for limited access to care, the incidence is 2,277 per 100,000 children < 5 years.

Since the signs and symptoms of Hib pneumonia cannot be differentiated from those of pneumonia caused by other microorganisms, the incidence of Hib pneumonia can best be approximated from Hib vaccine trials[18]. Meta-analysis of Hib vaccine trials in the Gambia and Indonesia showed that Hib causes approximately 5% (95% CI 3%–14%) of clinical pneumonia [6], [7]. This corresponds to Hib pneumonia incidences of 115 and 114 per 100,000 children < 5 years in Belarus and Uzbekistan, respectively. These rates are approximately half of the numbers reported by the GBD project, which estimated an average Hib pneumonia incidence in the WHO European region of 283 per 100,000 children < 5 years in the absence of Hib vaccine (range 259–463) [6]. This estimate is used in sensitivity analyses.

#### Hib NPNM

There are no data on Hib NPNM diseases in Belarus and Uzbekistan. Studies from Bulgaria and Czech Republic assessing all types of Hib diseases reported an average of 0.57 NPNM cases for each meningitis case [19], [20]. The most important type was epiglottitis, accounting for 78% of cases. We thus assumed a Hib NPNM incidence of 6.15 and 10.65 per 100,000 children < 5 years in Belarus and Uzbekistan, respectively (Table 1). The GBD project estimated a Hib NPNM incidence rate of 5.0 per 100,000 children < 5 years for the WHO European region [6].

### Case fatality ratios

In the Minsk city meningitis surveillance project, one of the 30 children with confirmed Hib meningitis died, giving a case fatality ratio of 3.2%. This is comparable to CFRs reported in Western European countries, such as 5% in the United Kingdom [21] and 4% in France [22]. Only six and five pneumonia deaths in children < 5 years were reported in Belarus during 2007 and 2008, respectively. This is equivalent to around 2% of all deaths in children and a CFR of 0.3% for all-cause pneumonia.

In Uzbekistan, 22 children < 5 years were reported to have died from meningitis in 2007, giving a CFR of 0.42%. For Hib meningitis, this figure is however unrealistically low. We used data from an Indian study reporting a hospital CFR of 15% [23] and assumed a CFR of 100% for the 32% of children that do not reach formal health care [24]. The overall CFR was thus 42%. According to Uzbek hospital surveillance data, a total of 214 pneumonia deaths occurred in 2007, generating all-cause hospital pneumonia CFR of 1.07%. We assumed 10% CFR for those without access to care, giving a weighted mean of 4%. NPNM CFRs were assumed similar to those of meningitis for the 78% epiglottitis proportion and zero for the other NPNM diseases (Table 1).

The GBD project estimated CFRs in the WHO European region of 5%, 27% and 1% for Hib pneumonia, meningitis and NPNM, respectively [6]. These estimates are averages of a very diverse region in terms of income and access to health services. Albeit these estimates are very different than ours, we included them in the sensitivity analyses to illustrate the impact a change in CFRs has on the result.

### Meningitis sequelae

In Belarus, all children that have suffered from meningitis are followed up every three months during the first year after discharge and annually for the next two years. Data from MCCIDH revealed that 12% of children with confirmed Hib meningitis suffered from disability at six months follow-up; 75% of these had reduced hearing. Due to lack of data in Uzbekistan we used a global meta-analysis estimate of 14.5% of all Hib meningitis survivors suffering from sequelae [2].

### Disability adjusted life years

The standard DALY formula and disability weights of 0.28 and 0.616 for pneumonia and meningitis, respectively, were used [25]. As there are no disability weights available for any of the NPNM diseases, the meningitis weight was used as an approximation, as epiglottitis has comparable severity to meningitis. The duration of episode was assumed similar to the average length of stay in hospital for all acute illnesses and life-long for meningitis sequelae. Proportions of the eight different types of sequelae were as follows [2], with the respective disability weights in parentheses [25]: Cognitive difficulties 7.6% (0.0777), seizure disorders 10.4% (0.0987), hearing loss 22.2% (0.2331), motor deficit 15.3% (0.3884), visual disturbance 3.5% (0.6), behavioural problems 14.6% (0.0777), clinical impairments 8.3% (0.4687), and multiple disabilities 18.1%. A weighted disability weight of 0.8571 was calculated for multiple impairments.

### Treatment costs

Data on drug usage and average length of stay in hospital were collected from a review of patient records at selected health facilities in both countries. Costs of drugs were collected from hospital pharmacies and the costs per hospital bed-day were provided by hospital administrators. In Belarus, meningitis treatment costs were collected at MCCIDH and pneumonia costs from Minsk Regional Hospital and Stolbtsy District Hospital. In Uzbekistan, treatment costs of meningitis were collected at the Infectious Disease Hospital #1 and pneumonia data at the Paediatrics Research Institute, the Children's Hospital #5 of Yunusobod District, and the Uchtepa District Children's Hospital. Household costs for drugs were approximated by assuming that prescribed drugs were purchased in local pharmacies. Discounted lifetime treatment costs of meningitis sequelae were approximated from a Russian study as US$ 32,660 for both countries [26].

In Belarus, the average length of stay in hospital was 21 days per meningitis episode and between 12 and 14 days for pneumonia. In this country it is required that children who have suffered from meningitis should be re-hospitalised for follow-up one month and again six months after the initial discharge. Out of 18 meningitis cases at MCCIDH, two were re-admitted three times, seven two times and nine one time as part of their follow-up. We thus assumed that all children with meningitis were re-hospitalised 1.6 times with an average length of stay of 9 days. We further assumed three outpatient visits per pneumonia and NPNM episodes and seven visits per meningitis episode [11]. In Uzbekistan, the average length of stay in hospital was 11 days for meningitis and there is no policy for re-admission. The average length of stay for pneumonia was nine days and we assumed two outpatient visits for each Hib disease hospital admission. Costs of outpatient visits were based on WHO estimates; US$ 9.21 per visit in Belarus and US$ 1.53 per visit in Uzbekistan [27].

### Hib vaccine assumptions

In Belarus, a ministerial order dictates a four-dose Hib vaccine schedule, following a WHO recommendation of a booster dose in countries where Hib disease frequently occurs in children above 18 months of age [28]. In Minsk City, the schedule is 3, 4, 5 and 18 months, and the combined diphtheria-tetanus-pertussis (DTP)-Hib vaccine is procured from Sanofi Pasteur at US$ 4.95 per dose. We assumed a similar price for the national analysis. In Uzbekistan, three doses of combined DTP-hepatitis B-Hib vaccine are given at 2, 3 and 4 months. In 2010 Unicef procured the vaccine for the GAVI Alliance at a price per dose of US$ 3.00[29]. Since both countries use a single dose vial, vaccine wastage is only estimated as 5%.

We interviewed vaccine programme staff to assess any logistical costs related to introducing the vaccine. It was revealed that even though vaccine volume had increased, existing cold chain capacities were sufficient in both countries with no additional investments required. In Uzbekistan, a GAVI introduction grant of US$ 100,000 was used for staff training and this cost was included in the analysis.

Vaccination coverage data reported by WHO and Unicef were used[30]. Hib vaccine efficacy was assumed as 86% after three doses [31], 81% after two doses [32] and 51% after one dose [32].

### Sensitivity analysis

To illustrate the impact of changes in incidence estimates and CFRs, the primary sensitivity analysis was a scenario using the WHO European region GBD numbers for both countries; thus assuming that the epidemiological parameters are similar in the two settings. Univariate sensitivity analyses were also done by assuming 100% access to care in Uzbekistan, a Hib vaccine price of US$ 2 per dose and no discounting of future values. Furthermore, since the decision-analytic model is static, indirect effects from lower risk of exposure in unvaccinated children, commonly referred to as “herd immunity”, are not directly incorporated[33]. To evaluate the potential importance of herd effects, we multiplied the direct vaccine impact by 20% in a scenario analysis [34].

## Results

### Disease impacts

For Belarus, it was estimated that the vaccine prevents 493 Hib disease cases and three deaths each year, while in Uzbekistan 3,072 cases and 341 deaths are averted annually. Four and 34 cases of meningitis sequelae are also prevented each year in Belarus and Uzbekistan, respectively. The results in discounted form are shown in Table 3. Deaths averted represent approximately 0.3% and 1.1% of total 2009 under-five mortality in Belarus and Uzbekistan, respectively.

**Table 3:** Estimated Hib disease and treatment costs in Belarus and Uzbekistan with and without Hib vaccine for the 2009 birth cohorts.

	Belarus			Uzbekistan		
	No vaccine	Vaccine	Prevented	No vaccine	Vaccine	Prevented
*Cases:*						
Hib pneumonia	504	95	409	2,994	608	2,387
Hib meningitis	44	8	36	492	100	392
Hib NPNM	27	5	22	280	57	223
Meningitis sequelae	5	1	4	41	8	33
*Deaths:*						
Hib pneumonia	1	0	1	120	24	95
Hib meningitis	1	0	1	207	42	165
Hib NPNM	1	0	1	92	19	74
*DALYs*	187	35	152	14,382	2,909	11,473
*No. of outpatient visits*	1,903	357	1,546	5,122	1,040	4,082
*No. of hospital admissions*	647	121	525	2,561	520	2,041
*Outpatient visit costs (US$):*						
Hib pneumonia	14,082	2,644	11,438	6,231	1,265	4,966
Hib meningitis	2,883	541	2,342	1,023	208	816
Hib NPNM	752	141	611	583	118	465
*Inpatient admission costs (US$):*						
Hib pneumonia	223,195	41,907	181,288	397,055	80,598	316,457
Hib meningitis	86,705	16,280	70,425	110,921	22,516	88,405
Hib NPNM	20,220	3,796	16,423	63,172	12,823	50,348
*Meningtis sequelae costs (US$)*	75,261	14,048	61,213	905,085	182,861	722,224

### Treatment costs averted

In Belarus, the average costs per bed-day excluding patient-specific costs, such as drugs and meals, were US$ 40 at MCCIDH and US$ 23 at Stolbtsy District Hospital. When multiplying by average length of stay and including patient-specific items, average meningitis treatment costs amounted to US$ 1,311 per acute episode and average pneumonia costs to US$ 413, US$ 448 and US$ 542 for district, regional and MCCIDH levels, respectively.

In Uzbekistan, costs per hospital bed-day were estimated as US$ 9.10. Average costs of treating meningitis amounted to US$ 238, US$ 257 and US$ 276 at district, regional and national hospital levels, respectively, and the cost per pneumonia patient was estimated as US$ 268 for all levels. Annual treatment costs with and without Hib vaccine are summarised in Table 3. In Uzbekistan, approximately 27% of the costs are covered by households as out-of-pocket payments for drugs.

### Incremental vaccine delivery costs

Without Hib vaccine, costs of vaccines and syringes per fully vaccinated child amounted to approximately US$ 23 in Belarus and US$ 8 in Uzbekistan (Table 4). Introduction of Hib vaccine increases annual costs by 84% in Belarus and 101% in Uzbekistan, leading to costs per fully vaccinated child of US$ 43 and US$ 16, respectively.

**Table 4:** Vaccine and syringe costs with and without Hib vaccine (2010 US$).

	Belarus				Uzbekistan			
Antigen	Doses in schedule	Vaccine costs	Injection supplies	Total	Doses in schedule	Vaccine costs	Injection supplies	Total
BCG	1	14,231	9,572	23,802	1	49,928	56,931	106,859
Hepatitis B	3	184,284	26,049	210,333	4	694,253	189,769	884,023
DTP	4	63,945	34,732	98,677	4	588,462	189,769	778,232
MMR	1	245,634	12,060	257,694	1	1,121,184	54,523	1,175,707
OPV	2	72,178	-	72,178	5	1,264,578	-	1,264,578
IPV	3	1,402,315	26,049	1,428,364	0	-	-	-
*Total without Hib vaccine*		1,982,586	108,462	2,091,049		3,718,406	490,993	4,209,399
Costs per child w/o Hib vaccine		21.85	1.20	23.04		7.05	0.93	7.98
Hib combination vaccine*	4	1,814,760	48,239	1,862,999	3	5,345,975	142,327	5,488,302
*Total with Hib vaccine* **		3,733,401	121,969	3,855,371		8,102,345	348,666	8,451,011
Costs per child with Hib vaccine		41.14	1.34	42.48		15.36	0.66	16.02
**Incremental costs**		**1,750,815**	**13,507**	**1,764,322**		**4,383,938**	**−142,327**	**4,241,611**

*DTP-Hib in Belarus and DTP-HepB-Hib in Uzbekistan.

**In Uzbekistan, hepatitis B vaccine birth dose and booster dose of DTP at 18 months are included here.

### Cost-effectiveness

Costs per discounted DALY averted amount to US$ 9,323 in Belarus and US$ 267 in Uzbekistan (Table 5). Since life years gained represent as much as 66% and 97% of incremental DALYs in Belarus and Uzbekistan, respectively, discounting makes a considerable difference to the ICER. Without discounting, the results are US$ 3,573 in Belarus and US$ 77 in Uzbekistan. When using the Uzbekistan GAVI co-financing amount of US$ 0.30 per dose instead of the vaccine price, Hib vaccine is cost saving for the Government. Each year, the Uzbek Government saves approximately US$ 6.18 million; 5 million for vaccines and syringes donated by GAVI and 1.18 million in treatment costs averted.

**Table 5:** Incremental cost-effectiveness of Hib vaccine for the 2009 birth cohort in Belarus and Uzbekistan: Base case analysis and alternative scenario using GBD EURO estimates (discounted values).

	Base case		With GBD EURO estimates*	
	Belarus	Uzbekistan	Belarus	Uzbekistan
Annual incremental vaccine costs (US$)	1,764,322	4,241,611	1,764,322	4,241,611
Treatment costs averted (US$)	343,740	1,183,681	676,114	1,676,923
Annual net costs (US$)	1,420,582	3,057,930	1,088,208	2,564,688
Hib disease cases averted	467	3,002	1,081	6,373
Hib deaths averted	3	334	66	388
Hib meningitis sequelae cases averted	4	33	3	36
DALYs averted	152	11,473	2,316	13,313
Incremental costs per death averted (US$)	485,567	9,162	16,514	6,606
Incremental costs per DALY averted (US$)	9,323	267	470	193

*Global Burden of Disease incidence and case fatality rates for the WHO European region [6].

### Sensitivity analysis

**Figure 2:**
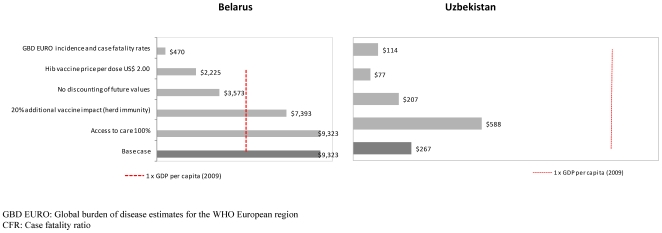
Scenario analysis. Impact on discounted costs per DALY averted from univariate changes in parameter values.

When using the GBD EURO parameter values for incidence and CFRs the vaccine becomes considerably more cost-effective in Belarus while there are only marginal differences in the Uzbekistan results (Table 5). Since the CFR for meningitis in the GBD EURO estimates is 27%, while we assumed 3.2% in the base case for Belarus, considerably more deaths are averted in the GBD scenario, increasing the cost-effectiveness of the vaccine. Increased access to care in Uzbekistan makes the vaccine less cost-effective as fewer deaths would be averted (Figure 2). In contrast, a reduction in the vaccine price to US$ 2.00 per dose would improve the cost-effectiveness markedly in both countries.

## Discussion

Our analysis demonstrated that the ICER is considerably less in Uzbekistan than in Belarus. The most important reason for this difference is that the baseline Hib mortality burden, expressed as case fatality rates, is higher in Uzbekistan than in Belarus, leading to more deaths averted per child vaccinated. Table 6 provides a comparison of key indicators on the two countries. It is seen that the less than five mortality rate is 17 times higher in Uzbekistan than in Belarus and that the proportion due to pneumonia is approximately 21% in Uzbekistan while only 4% in Belarus. Hence, Hib vaccine will prevent proportionally more deaths in Uzbekistan than in Belarus and since mortality is the most important driver of DALYs, the ICER becomes more favourable in Uzbekistan.

**Table 6:** Comparison of key indicators and study results between the two countries.

Indicator	Belarus	Uzbekistan	Source
2009 GDP per capita	US$ 5,560	US$ 1,100	[37]
Hospital beds per 1,000 population	11	5	[45]
2008 mortality rate per 100,000 children < 5 years	1,245	21,200	[46]
Percent of deaths due to pneumonia in children < 5 years	4%	21%	[46]
Percent of deaths in children < 5 years prevented from Hib vaccine	0.28%	1.1%	Present analysis
Incremental costs of Hib vaccine introduction per fully vaccinated child	US$ 19.44	US$ 8.04	Present analysis
Incremental costs per discounted DALY averted	US$ 9,323	US$ 267	Present analysis

Other, albeit less important, explanations for the ICER difference are that the vaccine price is higher in Belarus than in Uzbekistan and that Belarus uses a four-dose schedule while only three doses are used in Uzbekistan. Incremental vaccine costs per child are consequently US$ 21.17 in Belarus and only US$ 7.0 in Uzbekistan. A booster dose is recommended in countries where Hib disease is a substantial problem in children above 12 months [35]. In the Minsk city meningitis surveillance it was found that as many as 80% of Hib cases were above 12 months; confirming the need for a booster dose. While there is no age-specific data on Hib disease available from Uzbekistan, the requirement for a booster dose might be less in this country. In a review of Hib disease age distributions, Bennett *et al*. found that while 60% of cases were above 12 months in the WHO European, the average was only 20% in the WHO South East Asian region [36]. It is thus possible that a similar impact is achieved in Uzbekistan with three doses as with four doses in Belarus, but this hypothesis can only be confirmed by undertaking intensive Hib disease surveillance.

When deciding whether Hib vaccine is a good use of scarce resources, the ICER values need to be viewed in relation to ability and willingness to pay for health care. The 2009 Gross Domestic Products (GDP) per capita was US$ 5,560 in Belarus and US$ 1,100 in Uzbekistan[37]. Hence, in relative terms, the Belarus Government is able to pay considerably more per DALY averted than the Uzbekistan Government. According to WHO recommendations, interventions that cost less than GDP per capita should be considered highly cost-effective and those that cost between one and three times GDP per capita cost-effective[38]. Hence, with these thresholds Hib vaccine can be considered highly cost-effective in Uzbekistan and cost-effective in Belarus. While economic evaluations of other interventions are limited in the two countries, a study on rotavirus vaccine in Uzbekistan was published in 2007[39]. At a rotavirus vaccine price of US$ 5 per course, it was estimated that the costs per discounted DALY averted amount to between US$ 75 and US$ 242; values comparable with our findings for Hib vaccine.

Our results are in a similar range with other economic evaluations of Hib vaccine. In a systematic review it was found that, in 2008 values, the costs per discounted DALY averted amounted to US$ 42 in Kenya, US$ 69 in Indonesia and US$ 10,842 in Moscow [40]. The favourable cost-effectiveness ratios in Kenya and Indonesia can be attributed to higher infant mortality and Hib disease incidence rates in these parts of the world than seen in Eastern Europe and Central Asia [6]. Economic evaluations of Hib vaccine in high-income countries include studies from France [41], Sweden [42], [43] and the USA [44]. In the French study, the costs per discounted Quality Adjusted Life Year gained amounted to US$ 8,054, which is comparable to our result from Belarus. In the Swedish and the US studies a cost-benefit approach involving attaching a monetary value to life years lost to death and disability were used and it was concluded that the vaccine is cost saving in both countries.

The main limitation of our study is relatively large uncertainty in the disease-specific parameter values, in particular for pneumonia. This is a limitation faced by all researchers working within the field of invasive bacterial diseases in countries with laboratory resource constraints. In many settings, the true incidence of Hib disease remains largely unknown because the signs and symptoms are difficult to differentiate from those caused by other microorganisms, such as *Streptococcus pneumoniae*, viruses and parasites. Indeed, the true contribution from a particular bacterium can only be determined from high-quality laboratories, but these are still lacking in many places. Moreover, even in settings with adequate laboratory services, widespread use of antibiotics before hospitalization is an important constraint to detection as this hinders growth of the bacteria. Finally, in many low-income countries, children with the highest disease burden have poor access to care and many die before they reach hospital. We adjusted for this factor in Uzbekistan, but since the exact value of this proportion is very difficult to determine, this parameter is one of the most uncertain in our analysis.

An additional limitation is the use of a static model that does not reflect the change in the force of infection over time. However, data needed for a dynamic model, such as age-specific carriage rates and population mixing are not readily available from the two countries. We incorporated possible herd immunity effects in a crude manner in the scenario analysis, but this prediction can only be considered a best guess. However, it could be argued that from a policy perspective there is limited additional benefit of a dynamic analysis when a static model, which takes a conservative approach, already demonstrates cost-effectiveness.

To increase the strength of our analysis, we prioritised data from national surveillance systems, even if more reliable values might be available from large-scale epidemiological studies in other countries. Our analysis is thus an example of how to undertake an economic evaluation with data gathered from routine data sources while using results from other countries only as a quality marker. Undertaking the analysis for two countries simultaneously brings several advantages as useful comparisons can be made on both parameter values and the overall results. When assessing the marked differences in terms of costs per discounted DALY averted between the two countries, valuable insights into the most important drivers of the results were made. Moreover, a key limitation frequently stated regarding economic evaluation studies is that they cannot easily be compared because of differences in methodologies. We facilitated such a comparison by using the same decision-analytic model for the two countries.

While data on Hib disease burden from Eastern Europe and Central Asia remain scarce, we have illustrated that the vaccine can be considered cost-effective despite conservative assumptions about incidence and mortality. However, international cost-effectiveness thresholds say nothing about affordability, and with increases in vaccine costs of 84% and 101%, respectively, both Governments should consider carefully the long-term financial sustainability. The GAVI Alliance is currently increasing its support for pneumococcal and rotavirus vaccine introductions and it is likely that its support to Hib vaccine will stop when the current five year commitments come to an end. In countries with relatively high under-five mortality, such as Uzbekistan, Hib vaccine is a highly cost-effective intervention and it is therefore vital that the future financial sustainability of the vaccine is secured.
